# Tuina therapy for patients with chronic fatigue syndrome: a randomized controlled trial

**DOI:** 10.1186/s12967-025-07624-7

**Published:** 2026-01-08

**Authors:** Shoujian Wang, Jun Ren, Xin Zhou, Sitong Fang, Tianxiang He, Zhiwei Wu, Shanda Xu, Lingjun Kong, Min Fang

**Affiliations:** 1 Department of Tuina, Shuguang Hospital, Shanghai University of Traditional Chinese Medicine, Shanghai, China; 2 Yueyang Hospital of Integrated Traditional Chinese and Western Medicine, Shanghai University of Traditional Chinese Medicine, Shanghai, China; 3 Institute of Tuina, Shanghai Institute of Traditional Chinese Medicine, Shanghai, China

**Keywords:** Chronic fatigue syndrome, Tuina, Randomized controlled trial, Fatigue

## Abstract

**Background:**

Chronic fatigue syndrome (CFS) is a complex and disabling disorder characterized by persistent fatigue and functional impairment, with limited safe and effective nonpharmacologic treatment options. Tuina, a traditional manual therapy in Chinese medicine, has been increasingly applied for fatigue-related conditions, yet high-quality clinical evidence remains scarce. This study aimed to evaluate the efficacy and safety of Tuina combined with usual care (UC) in adults with CFS.

**Methods:**

This assessor-blinded, single-center randomized clinical trial was conducted from April 10, 2024, to January 10, 2025, at Shuguang Hospital, Shanghai University of Traditional Chinese Medicine. A total of 110 participants meeting the 1994 US Centers for Disease Control and Prevention diagnostic criteria for CFS were randomly assigned (1:1) to receive Tuina plus UC or UC alone. The Tuina group received three 20-minute sessions per week for 4 weeks (12 sessions in total) in addition to UC, which included health education, lifestyle guidance, and individualized symptom-targeted treatments but excluded Tuina therapy. The primary outcome was fatigue severity at 4 weeks measured by the 11-item Chalder Fatigue Questionnaire (CFQ-11). Secondary outcomes included anxiety and depression (Hospital Anxiety and Depression Scale), sleep quality (Pittsburgh Sleep Quality Index), and physical functioning and bodily pain (Short Form-36 subscales). Data were analyzed using analysis of covariance with baseline scores as covariates.

**Results:**

Baseline characteristics were comparable between groups. At week 4, compared with UC alone, Tuina plus UC led to greater improvement in fatigue (CFQ total score: adjusted mean difference −2.90 [95% CI −4.69 to −1.12]; *p* = 0.002; effect size 0.62), and more participants achieved the minimal clinically important difference in CFQ total score (89.1% vs 69.1%; *p* < 0.01). Benefits also favoured Tuina for physical fatigue (−2.21 [95% CI −3.40 to −1.02]; *p* < 0.001; effect size 0.70), whereas the effect on mental fatigue was smaller (−0.79 [95% CI −1.52 to −0.06]; *p* = 0.03; effect size 0.41). Tuina further reduced anxiety (−1.79 [95% CI −2.92 to −0.65]; *p* = 0.002) and improved overall sleep quality (PSQI total score: −1.62 [95% CI −2.60 to −0.65]; *p* = 0.0013); among PSQI subscales, only sleep duration remained statistically significant after Bonferroni correction. Other secondary outcomes did not differ significantly between groups, and no serious adverse events occurred.

**Conclusions:**

Tuina combined with UC produced clinically meaningful improvements in overall fatigue and significant improvements in physical fatigue compared with UC alone, with additional benefits in anxiety and overall sleep quality, and was safe and well tolerated. These findings suggest that Tuina may be a promising adjunctive therapy for CFS and warrant confirmation in larger multicenter trials with longer-term follow-up.

**Trial registration:**

International Traditional Medicine Clinical Trial Registry, ITMCTR2025000002. Registered 1 January 2025 – Retrospectively registered, https://itmctr.ccebtcm.org.cn/mgt/project/view/-5709466987144715200.

**Supplementary information:**

The online version contains supplementary material available at 10.1186/s12967-025-07624-7.

## Introduction

Chronic Fatigue Syndrome (CFS), also referred to as myalgic encephalomyelitis, is a complex and disabling condition characterized by persistent, unexplained fatigue lasting more than six months, often accompanied by musculoskeletal pain and marked impairments in daily functioning [1]. Globally, the prevalence of CFS is estimated at 0.4%–2.6%, with a higher incidence in women; epidemiological surveys in China have reported prevalence rates of 6.42% [2, 3]. The prognosis remains poor, with only a minority of patients achieving full recovery [4, 5]. Despite decades of investigation, diagnosis is still based on symptom-oriented criteria, and no curative treatment is available [6–9]. Consequently, CFS imposes a substantial and long-term burden on patients, families, and healthcare systems [1].

Current management of CFS primarily emphasizes non-pharmacological interventions, notably graded exercise therapy (GET) and cognitive behavioral therapy (CBT) [10–12]. Although some trials have reported improvements in fatigue and physical functioning, findings remain controversial [13]. In particular, the 2021 updated NICE guidelines explicitly withdrew recommendations for GET due to frequent reports of symptom exacerbation, while CBT is now considered only supportive rather than curative [13, 14]. These limitations highlight the urgent need for safe, effective, and accessible non-pharmacological treatments.

Tuina, a traditional Chinese manual therapy, has been increasingly applied to chronic fatigue and related functional disorders in China because of its favorable accessibility and holistic regulatory effects [15, 16]. In clinical practice, Tuina is delivered as a traditional Chinese medicine-based, prescription-driven intervention that integrates meridian- and acupoint-based pattern differentiation with standardized combinations of manipulative techniques (such as pressing, kneading, and grasping). Preliminary studies suggest potential benefits in reducing fatigue, improving sleep, and. enhancing quality of life [17]. However, most trials have combined Tuina with other therapies such as acupuncture or pharmacological treatments, leaving its therapeutic efficacy uncertain [18–20].

To address this evidence gap, we conducted an assessor-blinded randomized controlled trial to rigorously evaluate the efficacy and safety of Tuina for CFS. We hypothesized that Tuina plus usual care (UC) would yield superior improvements in fatigue and related symptoms compared with UC alone, while maintaining an acceptable safety profile.

## Methods

### Study design

We conducted an assessor-blinded, randomized controlled trial to evaluate the efficacy of Tuina plus UC versus UC alone in patients with CFS. The trial was designed and reported in accordance with the Consolidated Standards of Reporting Trials (CONSORT) guidelines for randomized controlled trials.

### Participants

Participants were recruited through both online advertisements (via WeChat) and offline posters displayed in the clinic. Individuals who expressed interest were invited to an outpatient screening visit, where eligibility was assessed by trained clinicians. Eligible participants who agreed to enroll provided written informed consent prior to study initiation. Baseline demographic information, including age, sex, occupation, height, and weight, was collected using standardized questionnaires (Table 1).

**Table 1:** Baseline characteristics of the intention-to-treat population

Characteristic	Tuina group （*n* = 55）	Control group （*n* = 55）	P value
Mean age (SD), years	34.78 (8.51)	34.87 (9.99)	0.865
Mean body mass index (SD), kg/m^2^	22.24 (2.95)	22.37 (2.83)	0.815
Gender, n (%)			
Male	17（30.9）	11（20.0）	0.189
Female	38（69.1）	44（80.0）	
Education Level, n (%)			
Bachelor’s degree or above	53（96.4）	50（90.9）	0.518
High school	0（0.0）	1（1.8）	
Junior college	2（3.6）	3（5.5）	
Middle school or below	0（0.0）	1（1.8）	
Occupation, n (%)			
Finance/Auditing personnel	1（1.8）	4（7.3）	0.149
Logistics personnel	0（0.0）	2（3.6）	
Technical/Research & Development personnel	12（21.8）	5（9.1）	
Teacher	2（3.6）	1（1.8）	
Student	11（20.0）	13（23.6）	
Customer service personnel	0（0.0）	2（3.6）	
Production personnel	0（0.0）	1（1.8）	
Sales personnel	1（1.8）	3（5.5）	
Marketing personnel	2（3.6）	1（1.8）	
Clerical/Administrative personnel	4（7.3）	5（9.1）	
Administrative personnel	1（1.8）	0（0.0）	
Professionals (e.g., accountants, lawyers, healthcare workers)	21（38.2）	15（27.3）	
Others	0（0.0）	3（5.5）	

Eligible participants were adults aged 18 years or older who met the diagnostic criteria for CFS according to the 1994 US Centers for Disease Control and Prevention (CDC) case definition, which was selected for this trial as it is widely applied in clinical practice in China [1]. The CDC criteria require persistent or relapsing fatigue lasting at least 6 months, not attributable to ongoing exertion, not substantially relieved by rest, and associated with a marked reduction in occupational, educational, social, or personal activities, together with at least four of the following eight symptoms present concurrently for at least 6 months: (1) self-reported impairment in short-term memory or concentration; (2) sore throat; (3) tender cervical or axillary lymph nodes; (4) muscle pain; (5) multi-joint pain without redness or swelling; (6) headaches of a new type, pattern, or severity; (7) unrefreshing sleep; and (8) post-exertional malaise lasting more than 24 hours. All participants underwent comprehensive clinical evaluation by trained physicians, including medical history, physical examination, mental state assessment, and laboratory testing, to exclude alternative medical explanations for fatigue.

Participants were excluded if they had current suicidal ideation, relevant psychiatric comorbidities, insufficient fluency in Mandarin Chinese, or inability to comprehend the study procedures.

### Randomization and masking

Randomization was performed centrally by an independent coordinator using a computer-generated block randomization sequence with randomly varying block sizes. Eligible participants were randomly assigned (1:1) to receive either Tuina plus UC (Tuina group) or UC alone (control group) after baseline assessment. Allocation concealment was ensured through sealed, opaque, sequentially numbered envelopes prepared in advance. Following written informed consent, each participant received an envelope that was opened in their presence to determine group allocation and initiate the assigned intervention.

The trial coordinator was responsible for randomization and informing participants and therapists of their assignments and was therefore not blinded. Given the nature of the intervention, participants and therapists could not be masked. However, outcome assessors and statisticians were blinded to group allocation throughout the study. In addition, both the Trial Steering Committee and the data monitoring board remained masked to treatment assignments.

### Procedures

Before randomization, all participants received at least one UC consultation provided by physicians experienced in the management of CFS. UC comprised health education, lifestyle guidance, and individualized symptom-targeted treatments, which could include pharmacotherapy, acupuncture, transcutaneous electrical nerve stimulation, or traditional Chinese medicine, but explicitly excluded Tuina therapy. Additional UC sessions were not routinely scheduled during the trial but remained available if deemed clinically necessary. UC was not standardized across participants.

Participants in the Tuina group received Tuina in addition to UC over a 4-week period (three sessions per week; 12 sessions in total), a duration aligned with previous manual-therapy trials and routine clinical practice for CFS [19, 21]. Each session lasted approximately 20 minutes and was administered by experienced Tuina practitioners who had completed standardized training and held certification in manual therapy. To ensure treatment consistency across participants, all practitioners adhered to a structured intervention protocol (Appendix S1 pp 1–2).

Participants were assessed at baseline and at 4 weeks after randomization. All outcomes were measured using self-reported questionnaires. Baseline assessments were conducted face-to-face at the outpatient clinic, whereas follow-up data were obtained either in person or via a secure online electronic system. No long-term follow-up was included in this trial.

## Outcomes

### Primary outcome

The primary outcome was the fatigue severity at 4 week after the intervention, assessed using the Chalder Fatigue Questionnaire (CFQ-11) [22]. The CFQ-11 is a validated self-report instrument widely applied in both clinical practice and research to evaluate fatigue severity and its impact [23]. It consists of 11 items rated on a 4-point Likert scale (0–3), yielding a total score ranging from 0 to 33, with higher scores indicating greater fatigue. Items 1–7 measure physical fatigue, whereas items 8–11 measure mental fatigue.

### Secondary outcomes

Secondary outcomes included:

- **Anxiety and depression**, assessed using the Hospital Anxiety and Depression Scale (HADS), which consists of 14 items (seven for anxiety and seven for depression), each scored from 0 to 3, with subscale scores ranging from 0 to 21 [24].
- **Sleep quality**, assessed by the Pittsburgh Sleep Quality Index (PSQI), comprising 19 items that yield a total score between 0 and 21, with higher scores indicating poorer sleep quality [25].
- **Physical functioning**, assessed by the Short-Form 36 Physical Function subscale (SF-36 PF), which consists of 10 items measuring physical activity limitations, with scores ranging from 0 to 100 (higher scores indicating better function) [26].
- **Bodily pain**, assessed by the SF-36 Bodily Pain subscale (SF-36 BP), with scores ranging from 0 to 100 (higher scores indicating less pain and less interference with daily life) [26].

### Safety outcomes

Adverse events (AEs) were self-reported by participants through follow-up questionnaires that included specific questions on any new health problems occurring during the intervention period. AEs were defined as any unfavorable or unintended medical occurrence during the study, irrespective of its relationship to the intervention. Serious adverse events (SAEs) were defined as events that were fatal, life-threatening, required hospitalization, resulted in persistent or significant disability, involved self-harm, or required medical intervention to prevent such outcomes. All SAEs were required to be reported to the principal investigator and the institutional ethics committee within 24 hours of recognition.

### Statistical analysis

The sample size calculation was based on the CFQ-11 total score as the primary outcome. We applied the standard formula for comparing two independent means [27]. In a previously published RCT in patients with CFS (*n* = 211), the mean between-group difference in CFQ-11 change scores was 4.10 points (SD 7.56), corresponding to a required sample size of 69 participants per group when assuming α = 0.05, power = 80%, and a 20% dropout rate [28]. Although the intervention in that study (graded exercise therapy) differed from Tuina, it provided one of the few robust external estimates of CFQ-11 variability in CFS populations.

In contrast, our pilot Tuina study showed a smaller observed variance (mean between-group difference 2.35 points; SD 3.32), yielding an estimated required sample size of 41 participants per group. Given the marked discrepancy between these estimates, and recognizing that the external estimate was derived from a different intervention while the pilot estimate came from a small sample, neither estimate alone was considered fully reliable. Therefore, using both as complementary references and taking recruitment feasibility into account, we targeted 55 participants per group (total *n* = 110).

Prior to hypothesis testing, the distribution of continuous variables (e.g., age, height, weight, BMI, and outcome scores) was assessed for normality using the Shapiro-Wilk test and for homogeneity of variance using Levene’s test [29]. Data not conforming to normality were analyzed using nonparametric methods.

The primary analysis was conducted in the intention-to-treat (ITT) population. Analysis of covariance (ANCOVA) was used to compare posttreatment scores between the Tuina and control groups at 4 weeks. For each outcome measure, the posttreatment score was entered as the dependent variable, the treatment group as the fixed factor, and the corresponding baseline score as the covariate. This method was applied to both the primary and secondary outcomes. Adjusted between-group mean differences at week 4 and their 95% confidence intervals were derived from these ANCOVA models. Model assumptions for ANCOVA (e.g., homogeneity of regression slopes, normality, and homoscedasticity of residuals) were tested prior to final model fitting. To facilitate clinical interpretation, we additionally estimated standardised mean differences (Cohen’s d) for the CFQ total score and its subscales at week 4, calculated as the adjusted between-group mean difference divided by the pooled SD at follow-up [30]. All statistical tests were two-sided, with *p* < 0.05 considered statistically significant; for PSQI subscales, *p* values were Bonferroni-adjusted for seven comparisons (α = 0.05/7).

In a secondary analysis, we compared the proportions of participants who achieved minimal clinically important difference (MCID) in fatigue, defined as a ≥ 3-point reduction in CFQ-11 total score, consistent with previous CFQ studies [28, 31]. Participants were dichotomized as responders (meeting the MCID threshold) or non-responders. Between-group differences in response rates were assessed using chi-square tests or Fisher’s exact tests, as appropriate.

Missing data on continuous variables were handled using multiple imputation with predictive mean matching (PMM), implemented via the mice package in R [32–34]. Five imputed datasets were generated separately by treatment group. Imputation models included age, sex, height, weight, education, and occupation. After performing multiple imputation, the imputed values were checked for logical consistency, ensuring that deterministic relationships, such as the total CFQ-11 score equaling the sum of the physical and mental subscale scores, were maintained.

Sensitivity analyses were conducted under both the ITT and per-protocol (PP) populations, the latter defined as participants who completed the intervention and provided outcome data. In additional sensitivity ANCOVA models for the primary outcomes, we further adjusted for sex, age, education level, and baseline anxiety and depression scores, in addition to the baseline CFQ values.

Safety analyses included all participants who completed follow-up assessments. AEs and SAEs were self-reported at 4 weeks and compared between groups using chi-square or Fisher’s exact tests.

### Data completeness and non-responder follow-up

If outcome data were incomplete, research staff attempted to contact participants by telephone or WeChat. If data were not returned after two attempts 1 week apart, follow-up was conducted via email or text message.

This trial is registered at International Traditional Medicine Clinical Trial Registry (ITMCTR), number ITMCTR2025000002.

## Results

**Fig. 1:**
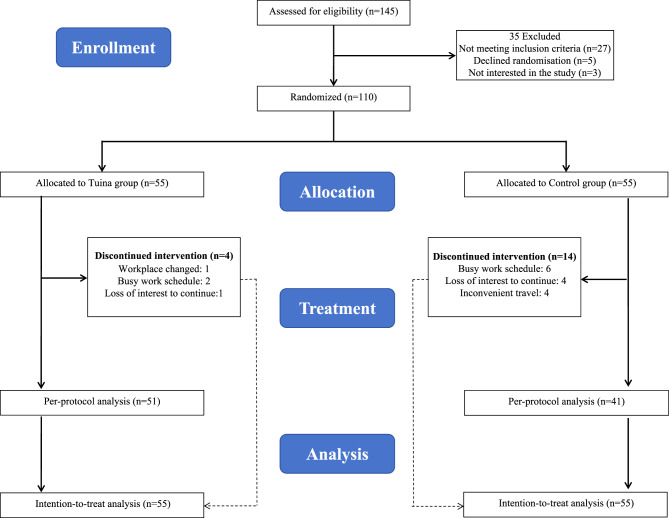
Trial profile

Between April 10, 2024 and January 10, 2025, a total of 362 patients were screened. Of these, 145 (40.2%) underwent eligibility assessment, and 110 (30.4%) were ultimately enrolled (Fig. 1). Among the 35 excluded patients, the most frequent reason was failure to meet the inclusion criteria (*n* = 27), followed by refusal to undergo randomization (*n* = 5) and lack of interest in study participation (*n* = 3). Subsequently, 55 patients were randomly assigned to the control group and 55 to the Tuina group. At 4 weeks, outcome missingness was 3 of 55 vs 14 of 55 for the CFQ-11 and 4 of 55 vs 14 of 55 for secondary outcomes (Tuina group vs control group).

Baseline characteristics of the participants are presented in Table 1. There were no significant differences between the Tuina and control groups in key demographic variables, including age (*p* = 0.865), BMI (*p* = 0.815), gender distribution (*p* = 0.189), educational attainment (*p* = 0.518), and occupational composition (*p* = 0.149), indicating comparable baseline profiles.

**Fig. 2:**
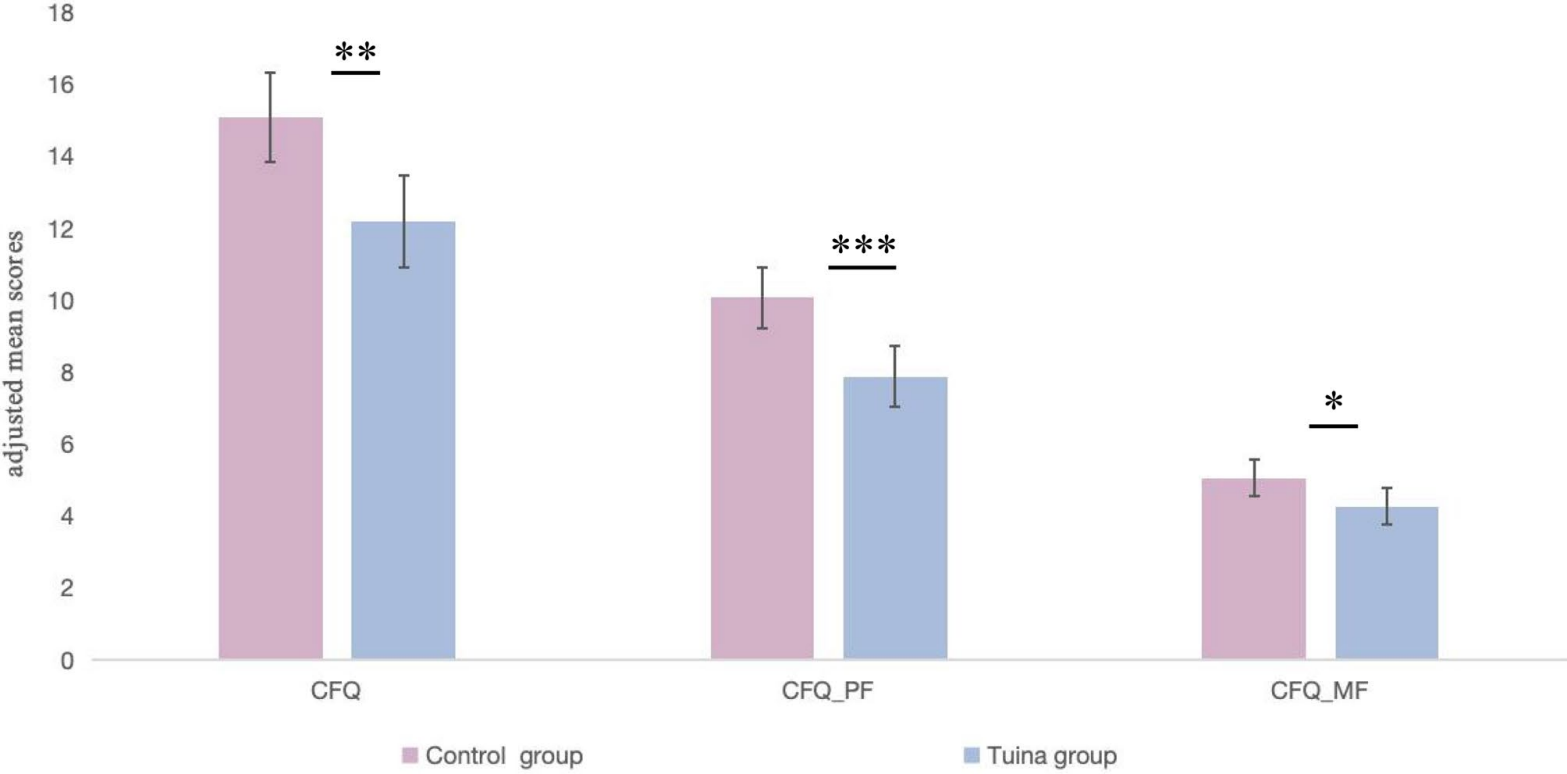
Adjusted mean Chalder fatigue Questionnaire (CFQ) scores at week 4. Bars represent least-squares mean scores for the total CFQ score and its physical (CFQ_PF) and mental (CFQ_MF) fatigue subscales at week 4 in the intention-to-treat (ITT) population, and error bars indicate 95% confidence intervals around the least-squares means. Least-squares means were estimated using analysis of covariance (ANCOVA) with the corresponding baseline score as a covariate. The ITT population comprised 55 participants in each group (Tuina and control). Significant between-group differences were observed for CFQ, CFQ_PF and CFQ_MF, with *p* values indicated as follows: ***p < 0.001; **p < 0.01; *p* < 0.05. CFQ, Chalder fatigue Questionnaire; CFQ_PF, CFQ physical fatigue subscale; CFQ_MF, CFQ mental fatigue subscale

**Fig. 3:**
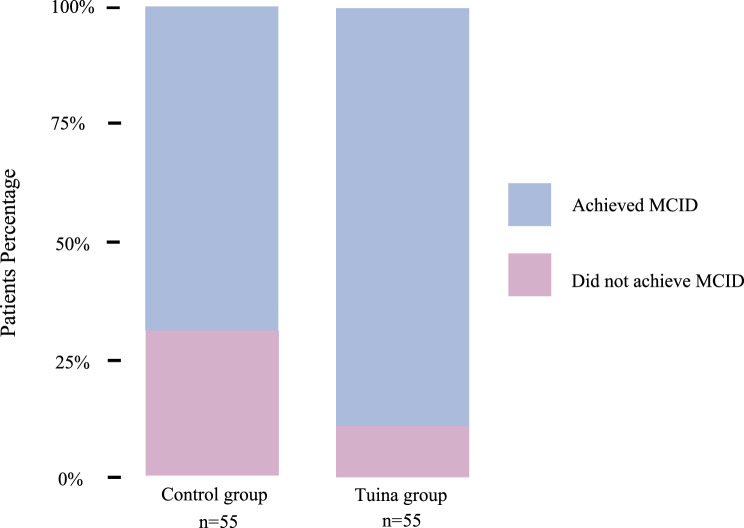
Proportion of participants achieving minimal clinically important difference (MCID) on CFQ from baseline to week 4. Stacked bars show the proportion of participants in the intention-to-treat (ITT) population in the Tuina group and control group who achieved a reduction of at least 3 points in Chalder fatigue Questionnaire (CFQ) scores from baseline (blue) versus those with less than 3 points reduction (pink). At week 4, a significant between-group difference was observed (*p* < 0.01) based on χ^2^ test. Numbers below the bars indicate the participants included in each group

Post-treatment outcome data collection was completed on January 15, 2025. Participants in the Tuina group showed significantly greater improvements in fatigue outcomes compared to the control group. After adjusting for baseline scores, the Tuina group demonstrated a mean difference of −2.90 (95% CI, −4.69 to −1.12; *p* = 0.002) on the CFQ total score, −2.21 (95% CI, −3.40 to −1.02; *p* < 0.001) on the physical fatigue subscale, and −0.79 (95% CI, −1.52 to −0.06; *p* = 0.03) on the mental fatigue subscale. The absolute effect sizes (Cohen’s d) were 0.62 for the CFQ total score, 0.70 for the physical fatigue subscale, and 0.41 for the mental fatigue subscale. These results indicate consistent benefits across multiple fatigue dimensions (Table 2; Fig. 2). Furthermore, 49 (89.1%) participants in the Tuina group showed an improvement of at least 3 points on the CFQ, compared to 38 (69.1%) in the control group (χ^2^ = 6.65; *p* < 0.01) (Fig. 3).

**Table 2:** Primary outcomes

	CFQ		CFQ_PF		CFQ_MF	
	Tuina group	Control group	Tuina group	Control group	Tuina group	Control group
**Baseline**						
n	55	55	55	55	55	55
Mean score (SD)	22.56 (4.43)	20.27 (5.19)	14.62 (2.97)	12.96 (3.40)	7.95 (2.01)	7.31 (2.21)
**4 weeks**						
n	55	55	55	55	55	55
Mean score (SD)	12.27 (4.37)	14.91 (4.81)	8.04 (2.85)	9.85 (3.36)	4.24 (1.93)	5.05 (1.87)
Adjusted mean difference compared with control group (95% CI)*	−2.90 (−4.69 to −1.12)	…	−2.21 (−3.40 to −1.02)	…	−0.79 (−1.52 to −0.06)	…
p value	0.002	…	< 0.001	…	0.03	…
Number improved from baseline†	49 (89.10%)	38 (69.10%)	…	…	…	…
Pearson χ^2^	6.65	…	…	…	…	…
p value	< 0.01	…	…	…	…	…

CFQ, Chalder Fatigue Questionnaire; CFQ_PF, Chalder Fatigue Questionnaire_physical faitgue subscale. CFQ_MF, Chalder Fatigue Questionnaire_mental fatigue. * Adjusted mean differences and 95% confidence intervals were estimated from ANCOVA models with the posttreatment score as the dependent variable, treatment group as the fixed factor, and the corresponding baseline score as the covariate. † By ≥ 3 points on the CFQ

Beyond fatigue, improvements were also observed in anxiety symptoms and sleep dimension at 4 weeks. Compared with the control group, the Tuina group reported lower levels of anxiety (MD, −1.79; 95% CI, −2.92 to −0.65; *p* = 0.002) and better overall sleep quality (MD, −1.62; 95% CI, −2.60 to −0.65; *p* = 0.0013), after adjusting for baseline scores. At 4 weeks, PSQI total scores were significantly lower in the Tuina group than in the control group, indicating better overall sleep quality. Among the PSQI subscales, the Tuina group showed greater improvement in sleep duration (adjusted mean difference −0.38, 95% CI −0.63 to −0.14; *p* = 0.002), and this difference remained statistically significant after Bonferroni correction. For the other PSQI subscales, between-group differences did not reach statistical significance after adjustment for multiple comparisons, although the point estimates generally favoured Tuina. Furthermore, no significant differences were found between groups in depressive symptoms, physical function, bodily pain. Full results are presented in Table 3 and Appendix S2 pp 3–5.

**Table 3:** Secondary outcomes

	Tuina group (n = 55)	Control group (n = 55)
**HADS-Anxiety scale**		
Mean score (SD) at baseline	8.27 (4.26)	7.58 (4.34)
Mean score (SD) at 4 weeks	5.45 (3.02)	6.96 (3.80)
Adjusted mean difference compared with control group (95% CI)*	−1.79 (−2.92 to −0.65)	…
p value	0.002	…
**HADS-Depression scale**		
Mean score (SD) at baseline	7.49 (3.82)	7.49 (3.82)
Mean score (SD) at 4 weeks	5.45 (3.58)	6.02 (3.53)
Adjusted mean difference compared with control group (95% CI)*	−0.56 (−1.75 to 0.62)	…
p value	0.347	…
**SF-36-Bodily pain scale**		
Mean score (SD) at baseline	45.33 (15.63)	49.80 (17.64)
Mean score (SD) at 4 weeks	55.40 (15.08)	56.80 (13.99)
Adjusted mean difference compared with control group (95% CI)*	−0.49 (−5.83 to 4.86)	…
p value	0.856	…
**SF-36-Physical functioning scale**		
Mean score (SD) at baseline	80.73 (18.34)	84.55 (13.69)
Mean score (SD) at 4 weeks	90.36 (10.40)	89.73 (10.65)
Adjusted mean difference compared with control group (95% CI)*	1.74 (−1.81 to 5.29)	…
p value	0.334	…
**PSQI_Total**		
Mean score (SD) at baseline	9.55 (3.08)	9.53 (3.28)
Mean score (SD) at 4 weeks	6.65 (2.21)	8.27 (3.26)
Adjusted mean difference compared with control group (95% CI)*	−1.62 (−2.60 to −0.65)	…
p value	0.0013	…
**PSQI_Sleep Duration**		
Mean score (SD) at baseline	1.04 (0.72)	1.15 (0.78)
Mean score (SD) at 4 weeks	0.76 (0.67)	1.16 (0.63)
Adjusted mean difference compared with control group (95% CI)*	−0.38 (−0.63 to −0.14)	…
p value	0.002	…
**PSQI_Sleep Disturbance**		
Mean score (SD) at baseline	1.42 (0.57)	1.35 (0.62)
Mean score (SD) at 4 weeks	1.25 (0.55)	1.47 (0.69)
Adjusted mean difference compared with control group (95% CI)*	−0.25 (−0.47 to −0.02)	…
p value	0.031	…
**PSQI_Daytime Dysfunction**		
Mean score (SD) at baseline	2.76 (0.43)	2.42 (0.69)
Mean score (SD) at 4 weeks	1.93 (0.77)	2.25 (0.89)
Adjusted mean difference compared with control group (95% CI)*	−0.42 (−0.74 to −0.10)	…
p value	0.011	…
**PSQI_Sleep Quality**		
Mean score (SD) at baseline	1.67 (0.72)	1.78 (0.71)
Mean score (SD) at 4 weeks	1.24 (0.54)	1.45 (0.69)
Adjusted mean difference compared with control group (95% CI)*	−0.20 (−0.43 to 0.03)	…
p value	0.092	…
**PSQI_Sleep Latency**		
Mean score (SD) at baseline	1.65 (1.08)	1.64 (1.01)
Mean score (SD) at 4 weeks	1.11 (0.85)	1.16 (0.83)
Adjusted mean difference compared with control group (95% CI)*	−0.06 (−0.34 to 0.21)	…
p value	0.656	…
**PSQI_Sleep Efficiency**		
Mean score (SD) at baseline	0.62 (0.76)	0.85 (1.11)
Mean score (SD) at 4 weeks	0.20 (0.59)	0.55 (1.00)
Adjusted mean difference compared with control group (95% CI)*	−0.27 (−0.56 to 0.02)	…
p value	0.067	…
**PSQI_Sleep Medication**		
Mean score (SD) at baseline	0.38 (0.78)	0.35 (0.89)
Mean score (SD) at 4 weeks	0.16 (0.46)	0.22 (0.69)
Adjusted mean difference compared with control group (95% CI)*	−0.07 (−0.24 to 0.10)	…
p value	0.427	…

*CFQ, Chalder Fatigue Questionnaire; HADS, Hospital Anxiety and Depression Scale, SF-36, 36-Item Short Form Health Survey, PSQI, Pittsburgh Sleep Quality Index. For the seven PSQI subscales, statistical significance was evaluated using a Bonferroni-corrected significance level (*α  = 0.05/7). * Adjusted mean differences and 95% confidence intervals were estimated from ANCOVA models with the posttreatment score as the dependent variable, treatment group as the fixed factor, and the corresponding baseline score as the covariate

Sensitivity analyses comprised two approaches. First, analyses were repeated in the PP population, yielding results that largely mirrored the primary ITT analysis: the between-group difference in CFQ total scores remained statistically significant, and similar patterns were observed for physical fatigue, anxiety, overall sleep quality (PSQI total score), and sleep duration, whereas differences in mental fatigue and other PSQI subscales were not significant but pointed in the same direction as the ITT findings (Appendix S3–S5, pp 6–13). Second, in ANCOVA models for the CFQ outcomes additionally adjusted for sex, age, education level, and baseline anxiety and depression scores (Appendix S4, p 9), the effects on CFQ total score and physical fatigue remained statistically significant and were similar in magnitude to those in the primary baseline-adjusted models, whereas the between-group difference in CFQ mental fatigue was attenuated and no longer statistically significant. Together, these analyses support the robustness of the main findings for overall and physical fatigue and selected secondary outcomes, while suggesting that the apparent benefit for mental fatigue is weaker and should be interpreted with greater caution.

No SAEs were reported in either the Tuina or control group during the 4-week intervention. Only minor, self-limiting reactions were observed in the Tuina group, with two participants experiencing transient local pain post-treatment, which resolved without medical intervention. None of the participants reported exacerbation of pre-existing conditions, nor was any medical attention required due to the trial procedures. These findings suggest that the Tuina intervention was generally safe and well tolerated by participants with CFS.

## Discussion

In this assessor-blinded RCT, Tuina plus UC led to greater improvements in overall fatigue than UC alone. Effects were more evident for the CFQ total score and the physical fatigue domain, and more patients in the Tuina group achieved the CFQ minimal clinically important difference. For mental fatigue, between-group differences tended to favour Tuina, but the effect was smaller. Beyond fatigue, Tuina produced significant reductions in anxiety symptoms and improved overall sleep quality on the PSQI, with sleep duration remaining significant after Bonferroni correction. These findings, which were broadly consistent in pp and covariate-adjusted sensitivity analyses, suggest that Tuina may provide a safe and potentially effective adjunctive therapy for patients with CFS.

For the secondary outcomes, Tuina significantly improved anxiety and global sleep quality, findings that are consistent with previous reports [17, 19]. In contrast, no significant between-group difference was observed in SF-36 physical function, which may be explained by a ceiling effect, as baseline scores were relatively high and left limited room for measurable improvement. Moreover, Tuina did not confer additional benefits over control in subjective sleep quality, sleep latency, sleep efficiency, use of sleep medication, or depressive symptoms. These null findings may reflect the relatively limited intensity and duration of the intervention, which may not have been sufficient to produce measurable improvements in these domains.

Current guidelines, including those from the National Institute for Health and Care Excellence (NICE), do not recommend the routine use of complementary therapies for CFS because of limited and inconsistent evidence [6]. Although several small clinical studies have evaluated Tuina for CFS, many have combined Tuina with other interventions or had methodological limitations, making it difficult to draw firm conclusions about its independent effects. Our assessor-blinded, single-center randomized trial comparing Tuina plus UC with UC alone therefore adds to the limited Tuina-specific evidence for CFS by using a standardized Tuina protocol, pre-specified endpoints, and validated measures of fatigue and function.

In our study, several outcomes that were significant in the ITT analysis (mental fatigue, sleep disturbance, and daytime dysfunction) lost statistical significance in the PP analysis, although the effect directions remained consistent. This concordance supports the robustness of the treatment effect, as both ITT and PP analyses pointed toward benefit, consistent with methodological guidance [35]. The loss of significance in PP is more likely attributable to reduced sample size and statistical power after excluding nonadherent participants, rather than a true absence of effect [36]. These findings underscore the reliability of the ITT results and highlight the need for larger, adequately powered trials to confirm efficacy among patients with higher adherence.

From a mechanistic perspective, several biological pathways have been hypothesized to contribute to the effects of Tuina on fatigue and related symptoms. Experimental and clinical studies suggest that Tuina and related manual therapies may influence neuroendocrine regulation and oxidative stress, for example through effects on tryptophan–serotonin metabolism and antioxidant capacity [37–39]. Such changes could, in principle, be relevant to fatigue, anxiety, and sleep in CFS. However, our trial did not include biomarker or neuroimaging assessments, and we therefore cannot draw any conclusions about specific biological mechanisms. Future studies should integrate longitudinal measurements of relevant biomarkers (e.g., tryptophan–serotonin metabolites, indices of oxidative stress) and brain imaging to directly test these mechanistic hypotheses in patients with CFS.

This study has several limitations. First, participants and practitioners were not blinded, which is a common challenge in trials of manual and other non-pharmacological interventions where sham procedures and double-blinding are often not feasible. In addition, all endpoints were patient-reported, without objective physiological or performance measures, which may increase the risk of bias. Second, this was a single-center trial conducted in a tertiary traditional Chinese medicine hospital in Shanghai, and the sample was predominantly female and relatively highly educated, which may limit generalizability to broader populations; in a post hoc analysis, however, sex did not appear to modify the treatment effect on the primary outcome (Appendix S6 pp 14). Third, follow-up was limited to the 4-week treatment period and dropout was higher in the control group, raising the possibility of attrition bias, although reasons for discontinuation were similar between groups, were not related to adverse events, and PP analyses yielded conclusions consistent with the ITT analyses.

In conclusion, this assessor-blinded RCT suggests that Tuina, when added to UC, provides clinically meaningful improvements in overall fatigue and significant improvements in physical fatigue in patients with CFS, with additional benefits in anxiety and selected aspects of sleep quality. These effects were broadly consistent across the ITT and sensitivity analyses, and Tuina was safe and well tolerated. Larger, multicentre trials with more diverse populations, longer-term follow-up, and objective outcome measures are needed to confirm these findings and further elucidate the underlying mechanisms.

## Electronic supplementary material

Below is the link to the electronic supplementary material.

Supplementary Material 1

Supplementary Material 2

## Data Availability

The analytical dataset can be obtained upon request by contacting the corresponding authors.
