# Supplementary material for: Tuina therapy for patients with chronic fatigue syndrome: a randomized controlled trial
Source: J Transl Med. 2026 Jan 8;24:301. doi: 10.1186/s12967-025-07624-7 (PMC12952015; doi:10.1186/s12967-025-07624-7)
Supplement: Supplementary file 2 — Supplementary Material 2 [file 12967_2025_7624_MOESM2_ESM.docx]

**Supplementary Materials**

[Appendix S1: Intervention Protocol 1](#_Toc1266442580)

[Appendix S1.1: Tuina Group 1](#_Toc1390016643)

[Appendix S1.2: Control Group 2](#_Toc1682606835)

[Appendix S2: Bar Chart of Secondary Outcomes (ITT Analysis) 3](#_Toc1508412149)

[Appendix S2.1: HADS 3](#_Toc838535408)

[Appendix S2.2: SF-36 4](#_Toc1821168068)

[Appendix S2.3: PSQI 5](#_Toc287298185)

[Appendix S3: Sensitivity analyses (PP population) 6](#_Toc1077356839)

[Appendix S4: Sensitivity analyses (additional covariate adjustment) 9](#_Toc1701765216)

[Appendix S5: Bar Chart of PP Analysis 10](#_Toc1380774566)

[Appendix S5.1 : CFQ 10](#_Toc969841280)

[Appendix S5.2 : HADS 11](#_Toc721512230)

[Appendix S5.3 : SF-36 12](#_Toc1830608336)

[Appendix S5.4 : PSQI 13](#_Toc1017654027)

[Appendix S6: Post-hoc analysis 14](#_Toc1151467081)

# Appendix S1: Intervention Protocol

## Appendix S1.1: Tuina Group

The Tuina intervention will be administered by five licensed Tuina practitioners. To ensure standardization of the treatment, four training sessions (each lasting 2 hours) will be conducted over a 2-week period. The training will consist of lectures, video demonstrations, and mutual hands-on practice. At the end of the training, an assessment will be conducted, and only practitioners who pass the assessment will be qualified to participate in the intervention. Both the training and assessment will be supervised by experts.

The acupoint selection and manipulation standards will follow the relevant content of Tuina (10th Edition), the national planning textbook for higher education institutions of Traditional Chinese Medicine.

**Treatment procedures**

***Head and face***: With the patient in the supine position, the practitioner applies pressing-kneading from Yintang (EX-HN3) to Shenting (GV24) for 30 seconds, followed by pushing manipulation from Yintang along the bilateral supraorbital ridges to the temples (Taiyang, EX-HN5) for 30 seconds. Pressing-kneading is then performed at Yintang (EX-HN3), Taiyang (EX-HN5), Baihui (GV20), Sishencong (EX-HN1), and Fengchi (GB20) for 4 minutes.

***Upper limbs***: With the patient in the supine position, the practitioner applies pushing manipulation along the medial side of the upper limb from proximal to distal, three times on each side, followed by pressing-kneading at Neiguan (PC6) bilaterally, for a total of 2 minutes.

***Abdomen***: With the patient in the supine position and both legs flexed, the practitioner performs clockwise abdominal rubbing for 1 minute.

***Lower limbs***: With the patient in the supine position, the practitioner applies pushing manipulation along the medial side of the lower limb from distal to proximal, repeated three times, followed by pressing-kneading at Sanyinjiao (SP6) bilaterally, for 2 minutes.

***Neck***: With the patient in the prone position, the practitioner performs pressing-kneading three times and pushing three times along the bilateral shoulders from the midline of the spine to the acromion, for a total of 2 minutes.

***Back and lower limbs***: With the patient in the prone position, the practitioner applies pressing-kneading along the Bladder meridian on both sides of the spine down to the heels, focusing on Feishu (BL13), Xinshu (BL15), Ganshu (BL18), Pishu (BL20), Shenshu (BL23), Weizhong (BL40), and Chengshan (BL57), repeated three times. This is followed by downward pushing along both sides of the spine to the heels, repeated three times, for a total of 8 minutes.

**Treatment regimen**

The pressing-kneading frequency will be maintained at 30 times per minute, with an average manual stimulation force of 3.0 kg. Each treatment session will last 20 minutes, performed three times per week, for a total of 12 sessions.

## Appendix S1.2: Control Group

Symptomatic treatment for chronic fatigue syndrome (CFS) generally includes dietary adjustment, pharmacological therapy, and appropriate exercise, with specific approaches as follows:

a. Dietary adjustment: Patients are advised to regulate their diet in a timely manner, avoid spicy and irritating foods as well as high-fat foods, and consume fresh vegetables and fruits in moderation, which may help eliminate toxins from the body and improve the condition.

b. Symptomatic treatment: Based on patients’ clinical manifestations and treatment needs, physicians may provide appropriate therapies such as medications, acupuncture, or transcutaneous electrical nerve stimulation (TENS) to control symptoms and promote recovery.

c. Appropriate exercise: Patients are encouraged to engage in suitable physical activities such as jogging or swimming according to their physical condition. However, if post-exertional malaise or fatigue occurs after exercise, this approach is not recommended.

The treatment will last for 4 weeks.

# Appendix S2: Bar Chart of Secondary Outcomes (ITT Analysis)

## Appendix S2.1: HADS

#
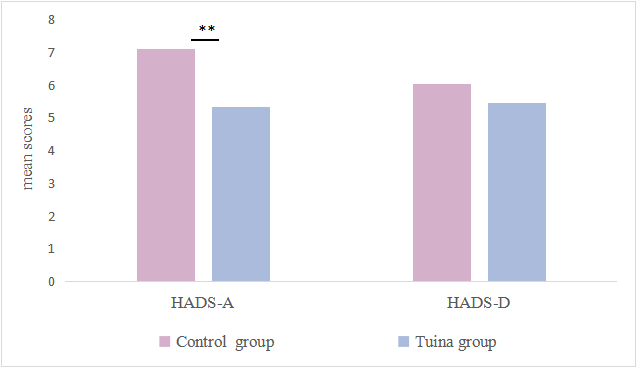


*Mean adjusted scores of HADS at week 4 in the intention-to-treat (ITT) population. Bars indicate least-squares mean scores of HADS-A and HADS-D for the Tuina group and the Control group, estimated using analysis of covariance (ANCOVA) with baseline scores as covariates. A significant between-group difference was observed in HADS-A. HADS-A, Hospital Anxiety and Depression Scale-Anxiety subscale; HADS-D, Hospital Anxiety and Depression Scale-Depression subscale; ** p < 0.01.*

## Appendix S2.2: SF-36


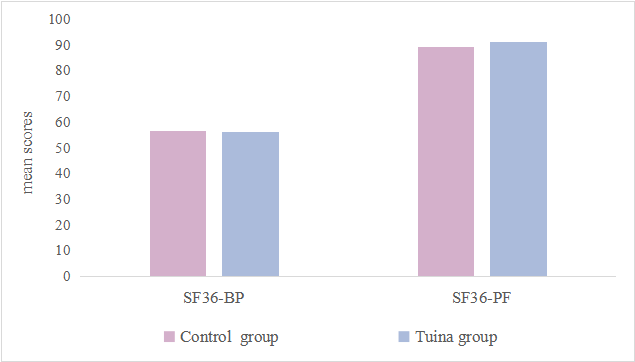


*Mean adjusted scores of SF-36 at week 4 in the intention-to-treat (ITT) population. Bars indicate least-squares mean scores of the Bodily Pain subscale (SF-36 BP) and Physical Functioning subscale (SF-36 PF) for the Tuina group and the Control group, estimated using analysis of covariance (ANCOVA) with baseline scores as covariates. No significant between-group differences were observed. SF-36 BP, Short Form-36 Health Survey–Bodily Pain; SF-36 PF, Short Form-36 Health Survey–Physical Functioning.*

## Appendix S2.3: PSQI


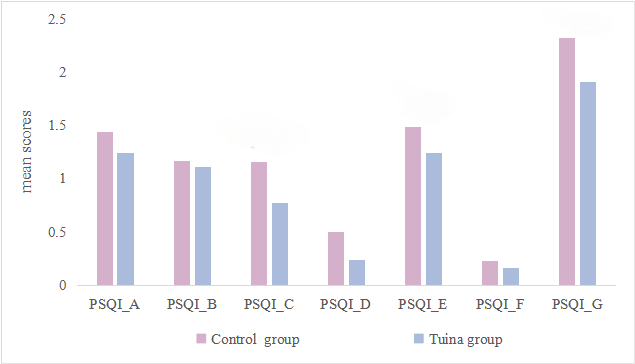


*Mean adjusted scores of the Pittsburgh Sleep Quality Index (PSQI) subscales at week 4 in the intention-to-treat*

*(ITT) population. Bars indicate least-squares mean scores of the seven PSQI subscales for the Tuina group and the Control group, estimated using analysis of covariance (ANCOVA) with baseline scores as covariates. Significant between-group differences were observed in PSQI_C, PSQI_E, and PSQI_G. PSQI_A, Sleep Quality; PSQI_B, Sleep Latency; PSQI_C, Sleep Duration; PSQI_D, Sleep Efficiency; PSQI_E, Sleep Disturbance; PSQI_F, Sleep Medication; PSQI_G, Daytime Dysfunction.*Statistical significance was evaluated using a Bonferroni-corrected significance level (α= 0.05/7≈ 0.007)*

# Appendix S3: Sensitivity analyses (PP population)

|  | **Tuina group** | **Control group** |
| --- | --- | --- |
| **CFQ-total** |  |  |
| n | 52 | 41 |
| Mean score (SD) at baseline | 22.69 (4.28) | 19.41 (4.68) |
| Mean score (SD) at 4 weeks | 11.94 (4.26) | 13.68 (4.95) |
| Adjusted mean difference compared with Control group (95% CI)* | -2.20 (-4.23 to -0.17) | ... |
| p value | 0.034 | ... |
| Number improved from baseline† | 47 | 29 |
| Pearson χ² | 5.93 | ... |
| p value | 0.015 | ... |
| **CFQ-physical fatigue scale** |  |  |
| n | 52 | 41 |
| Mean score (SD) at baseline | 14.69 (2.90) | 12.39 (3.10) |
| Mean score (SD) at 4 weeks | 7.87 (2.84) | 8.85 (3.28) |
| Adjusted mean difference compared with Control group (95% CI)* | -1.60 (-2.92 to -0.27) | ... |
| p value | 0.019 | ... |
| **CFQ-mental fatigue scale** |  |  |
| n | 52 | 41 |
| Mean score (SD) at baseline | 8.00 (1.97) | 7.02 (1.99) |
| Mean score (SD) at 4 weeks | 4.08 (1.87) | 4.83 (2.10) |
| Adjusted mean difference compared with Control group (95% CI)* | -0.76 (-1.60 to 0.09) | ... |
| p value | 0.081 | ... |
| **HADS-Anxiety scale** |  |  |
| n | 51 | 41 |
| Mean score (SD) at baseline | 8.18 (4.32) | 7.29 (4.47) |
| Mean score (SD) at 4 weeks | 5.37 (3.11) | 6.68 (4.26) |
| Adjusted mean difference compared with Control group (95% CI)* | -1.70 (-3.02 to -0.38) | ... |
| p value | 0.012 | ... |
| **HADS-Depression scale** |  |  |
| n | 51 | 41 |
| Mean score (SD) at baseline | 7.41 (3.69) | 7.85 (4.03) |
| Mean score (SD) at 4 weeks | 5.24 (3.63) | 6.44 (4.00) |
| Adjusted mean difference compared with Control group (95% CI)* | -0.99 (-2.37 to 0.40) | ... |
| p value | 0.160 | ... |
| **SF-36-Bodily pain scale** |  |  |
| n | 51 | 41 |
| Mean score (SD) at baseline | 45.57 (14.84) | 50.73 (16.05) |
| Mean score (SD) at 4 weeks | 55.65 (15.04) | 57.34 (14.81) |
| Adjusted mean difference compared with Control group (95% CI)* | 0.03 (-5.75 to 5.82) | ... |
| p value | 0.991 | ... |
| **SF-36-Physical functioning scale** |  |  |
| n | 51 | 41 |
| Mean score (SD) at baseline | 81.27 (18.78) | 85.00 (12.94) |
| Mean score (SD) at 4 weeks | 90.10 (10.61) | 89.51 (11.72) |
| Adjusted mean difference compared with Control group (95% CI)* | 1.89 (-2.09 to 5.87) | ... |
| p value | 0.348 | ... |
| **PSQI_Total** |  |  |
| n | 51 | 41 |
| Mean score (SD) at baseline | 9.49 (3.04) | 9.54 (3.45) |
| Mean score (SD) at 4 weeks | 6.55 (2.27) | 8.02 (3.75) |
| Adjusted mean difference compared with Control group (95% CI)* | -1.46 (-2.60 to -0.32) | ... |
| p value | 0.013 | ... |
| **PSQI_Sleep Duration** |  |  |
| n | 51 | 41 |
| Mean score (SD) at baseline | 1.04 (0.72) | 1.15 (0.82) |
| Mean score (SD) at 4 weeks | 0.82 (0.65) | 1.22 (0.73) |
| Adjusted mean difference compared with Control group (95% CI)* | -0.38 (-0.66 to -0.09) | ... |
| p value | 0.010 | ... |
| **PSQI_Sleep Disturbance** |  |  |
| n | 51 | 41 |
| Mean score (SD) at baseline | 1.41 (0.57) | 1.32 (0.65) |
| Mean score (SD) at 4 weeks | 1.27 (0.57) | 1.29 (0.72) |
| Adjusted mean difference compared with Control group (95% CI)* | -0.06 (-0.31 to 0.19) | ... |
| p value | 0.655 | ... |
| **PSQI_Daytime Dysfunction** |  |  |
| n | 51 | 41 |
| Mean score (SD) at baseline | 2.78 (0.42) | 2.39 (0.70) |
| Mean score (SD) at 4 weeks | 1.84 (0.73) | 2.00 (0.89) |
| Adjusted mean difference compared with Control group (95% CI)* | -0.31 (-0.66 to 0.03) | ... |
| p value | 0.074 | ... |
| **PSQI_Sleep Quality** |  |  |
| n | 51 | 41 |
| Mean score (SD) at baseline | 1.69 (0.74) | 1.76 (0.70) |
| Mean score (SD) at 4 weeks | 1.18 (0.52) | 1.27 (0.71) |
| Adjusted mean difference compared with Control group (95% CI)* | -0.07 (-0.32 to 0.17) | ... |
| p value | 0.552 | ... |
| **PSQI_Sleep Latency** |  |  |
| n | 51 | 41 |
| Mean score (SD) at baseline | 1.61 (1.08) | 1.56 (1.05) |
| Mean score (SD) at 4 weeks | 1.04 (0.85) | 1.22 (0.96) |
| Adjusted mean difference compared with Control group (95% CI)* | -0.20 (-0.52 to 0.11) | ... |
| p value | 0.201 | ... |
| **PSQI_Sleep Efficiency** |  |  |
| n | 51 | 41 |
| Mean score (SD) at baseline | 0.65 (0.89) | 0.93 (1.17) |
| Mean score (SD) at 4 weeks | 0.22 (0.61) | 0.73 (1.10) |
| Adjusted mean difference compared with Control group (95% CI)* | -0.42 (-0.75 to -0.09) | ... |
| p value | 0.014 | ... |
| **PSQI_Sleep Medication** |  |  |
| n | 51 | 41 |
| Mean score (SD) at baseline | 0.31 (0.74) | 0.44 (1.00) |
| Mean score (SD) at 4 weeks | 0.18 (0.48) | 0.29 (0.78) |
| Adjusted mean difference compared with Control group (95% CI)* | -0.06 (-0.26 to 0.14) | ... |
| p value | 0.557 | ... |

*CFQ, Chalder Fatigue Questionnaire; HADS, Hospital Anxiety and Depression Scale, SF-36, 36-Item Short Form*

*Health Survey, PSQI, Pittsburgh Sleep Quality Index. For the seven PSQI subscales, statistical significance was*

*evaluated using a Bonferroni-corrected significance level (α = 0.05/7). * Adjusted mean differences and 95%*

*confidence intervals were estimated from ANCOVA models with the posttreatment score as the dependent variable,*

*treatment group as the fixed factor, and the corresponding baseline score as the covariate. †By ≥3 points on the*

*CFQ.*

# Appendix S4: Sensitivity analyses (additional covariate adjustment)

|  | **CFQ** | | **CFQ_PF** | | **CFQ_MF** | |
| --- | --- | --- | --- | --- | --- | --- |
|  | **Tuina group** | **Control group** | **Tuina group** | **Control group** | **Tuina group** | **Control group** |
| **4 weeks** |  |  |  |  |  |  |
| n | 55 | 55 | 55 | 55 | 55 | 55 |
| Mean score (SD) | 12.27 (4.37) | 14.91 (4.81) | 8.04 (2.85) | 9.85 (3.36) | 4.24 (1.93) | 5.05 (1.87) |
| Adjusted mean difference compared with control group (95% CI)* | -2.63 (-4.46 to -0.80) | ... | -2.07 (-3.30 to -0.84) | ... | -0.66 (-1.41 to 0.09) | ... |
| p value | 0.005 | ... | 0.001 | ... | 0.09 | ... |

*CFQ, Chalder Fatigue Questionnaire; CFQ_PF, Chalder Fatigue Questionnaire_physical faitgue subscale. CFQ_MF, Chalder Fatigue Questionnaire_mental fatigue. * Adjusted mean differences and 95% confidence intervals were estimated from ANCOVA models with the posttreatment score as the dependent variable, treatment group as the fixed factor, and the corresponding baseline score, age, sex, education level, baseline depression score, and baseline anxiety score as covariates. † By ≥3 points on the CFQ.*

# Appendix S5: Bar Chart of PP Analysis

## Appendix S5.1 : CFQ


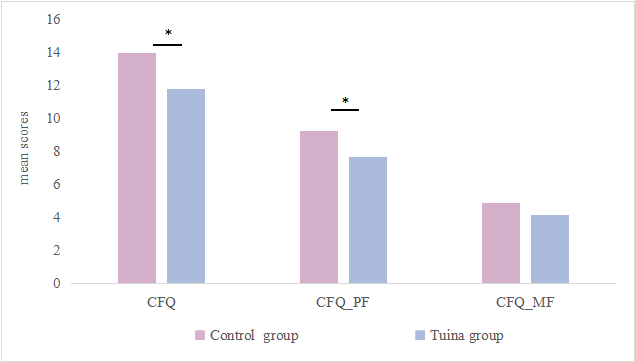


*Mean adjusted scores of CFQ at week 4 in the Per Protocol (PP) population. Bars indicate least-squares mean scores of CFQ, CFQ_PF and CFQ_MF for the Tuina group and the Control group, estimated using analysis of covariance (ANCOVA) with baseline scores as covariates. A significant between-group difference was observed in CFQ and CFQ_PF. CFQ, Chalder Fatigue Questionnaire; CFQ_PF, Chalder Fatigue Questionnaire_physical faitgue subscale. CFQ_MF, Chalder Fatigue Questionnaire_mental fatigue; * p < 0.05.*

## Appendix S5.2 : HADS

##
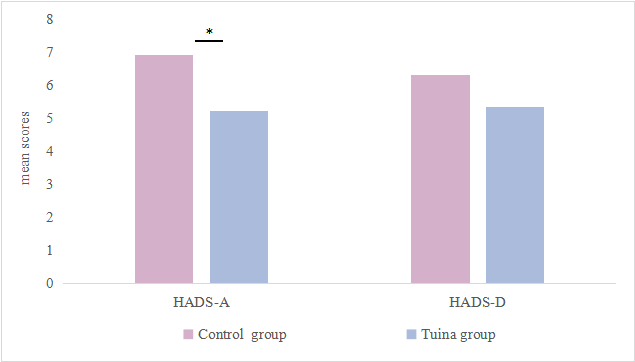


*Mean adjusted scores of HADS at week 4 in the Per Protocol (PP) population. Bars indicate least-squares mean scores of HADS-A and HADS-D for the Tuina group and the Control group, estimated using analysis of covariance (ANCOVA) with baseline scores as covariates. A significant between-group difference was observed in HADS-A. HADS-A, Hospital Anxiety and Depression Scale-Anxiety subscale; HADS-D, Hospital Anxiety and Depression Scale-Depression subscale; * p < 0.05.*

##

## Appendix S5.3 : SF-36

##
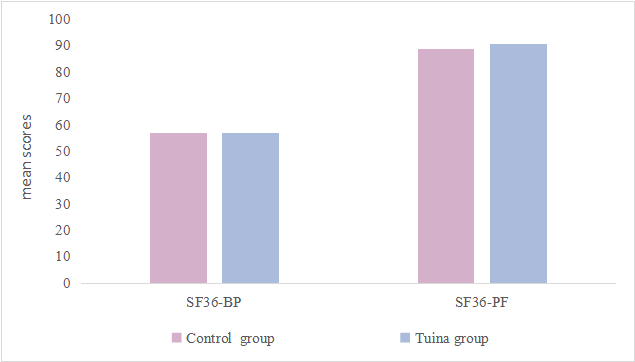


*Mean adjusted scores of SF-36 at week 4 in the Per Protocol (PP) population. Bars indicate least-squares mean scores of the Bodily Pain subscale (SF-36 BP) and Physical Functioning subscale (SF-36 PF) for the Tuina group and the Control group, estimated using analysis of covariance (ANCOVA) with baseline scores as covariates. No significant between-group differences were observed. SF-36 BP, Short Form-36 Health Survey–Bodily Pain; SF-36 PF, Short Form-36 Health Survey–Physical Functioning.*

## Appendix S5.4 : PSQI


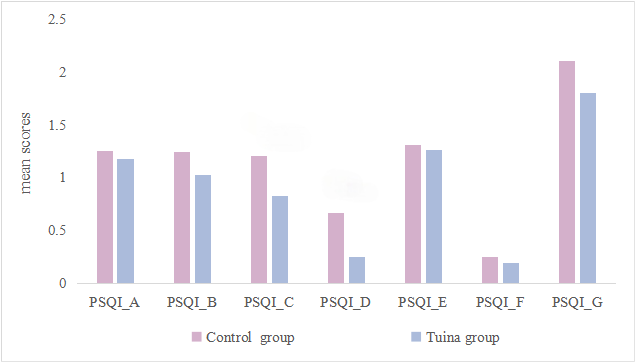


*Mean adjusted scores of the Pittsburgh Sleep Quality Index (PSQI) subscales at week 4 in the intention-to-treat*

*(ITT) population. Bars indicate least-squares mean scores of the seven PSQI subscales for the Tuina group and the Control group, estimated using analysis of covariance (ANCOVA) with baseline scores as covariates. Significant between-group differences were observed in PSQI_C, PSQI_E, and PSQI_G. PSQI_A, Sleep Quality; PSQI_B, Sleep Latency; PSQI_C, Sleep Duration; PSQI_D, Sleep Efficiency; PSQI_E, Sleep Disturbance; PSQI_F, Sleep Medication; PSQI_G, Daytime Dysfunction.*Statistical significance was evaluated using a Bonferroni-corrected significance level (α= 0.05/7≈ 0.007)*

# Appendix S6: Post-hoc analysis

| **Outcome** | **Interaction Term** | **β (95% CI)** | **P value** |
| --- | --- | --- | --- |
| CFQ | Group × Sex | -2.82 (-6.88 to 1.24) | 0.171 |

*CFQ, Chalder Fatigue Questionnaire*
